# Day Ward Glaucoma Patients Have Lower Depression Levels and Higher Glaucoma Knowledge Levels than Inpatients

**DOI:** 10.1155/2019/4182030

**Published:** 2019-09-08

**Authors:** Huiming Xiao, Wenmin Huang, Xi Qin, Chengguo Zuo, Qiongman Yang, Rong Li, Mingkai Lin

**Affiliations:** State Key Laboratory of Ophthalmology, Zhongshan Ophthalmic Center, Sun Yat-sen University, Guangzhou, China

## Abstract

**Importance:**

Psychological factors and glaucoma knowledge are closely related to the effects of glaucoma treatment.

**Background:**

Studies comparing anxiety and depression levels and glaucoma knowledge between glaucoma day-case patients and inpatients are limited.

**Design:**

Randomized clinical trial.

**Participants:**

Consecutive patients undergoing surgery were prospectively enrolled.

**Methods:**

Patients were randomized into the day-case group or the inpatient group. All of the patients underwent corresponding procedures for treatment, care, and education. All participants were asked to complete the General Condition Questionnaire, the Hospital Anxiety and Depression Scale (HADS), and the Gray Glaucoma Knowledge Questionnaire (GGKQ) at admission and the HADS and GGKQ at discharge.

**Main Outcome Measures:**

The scores for the General Condition Questionnaire, the HADS, and the GGKQ.

**Results:**

In total, 216 patients were enrolled in this study, including 119 day ward patients and 97 inpatients. There were no significant differences between the two groups in terms of their baseline demographic and clinical data (*P* > 0.05). The baseline HADS-anxiety (HADS-A), HADS-depression (HADS-D), and GGKQ scores were similar in both groups (*P* > 0.05). Before discharge, the difference in HADS-A scores between the two groups was not significant; however, the HADS-D scores of the day-case inpatients were significantly lower (*α* = 0.05, *P* < 0.001), and the GGKQ scores of day-case inpatients were significantly higher than those of the inpatients before discharge (*α* = 0.05, *P* < 0.001).

**Conclusions and Relevance:**

Day ward patients had lower levels of depression and higher levels of glaucoma knowledge.

## 1. Introduction

Glaucoma comprises a group of clinical syndromes or eye diseases that threaten the optic nerve and visual pathway and ultimately lead to visual impairment, mainly due to pathological ocular hypertension. While primary glaucoma is one of the most important psychosomatic ophthalmic diseases [1], personality, behavioural characteristics, and psychosocial factors are closely related to the occurrence and development of this disease [2, 3]. Moreover, the hospitalization process can become a type of psychological stimulation for patients [4].

Inpatient care is a traditional treatment pattern requiring hospitalization for 24 hours or longer [5], while day ward care only requires 1 working day [6]. Day ward care is an efficient medical service model that is conducive to decreasing inefficiencies in terms of time spent in the hospital, thus effectively relieving the patient backlog, increasing the bed occupancy rate, reducing the economic burden of patients, and alleviating doctor-patient conflicts. Compared with outpatient visits, day wards help patients enjoy health care at public expense and reserves reimbursement for those who need to be hospitalized

Day surgery has a history over one hundred years long; it was first reported in 1909 by James Nicholas, a British surgeon. It has been widely carried out in the United States, Canada, Britain, Singapore, Japan, and other countries and accounts for 70% of surgeries performed in European and American countries [7]. On the contrary, day surgery has only a ten-year history in China and comprises only 20% of the overall proportion of operations [8]. Ophthalmic surgery, which carries a low anaesthesia risk, requires a short operative time, is performed on patients who are typically in good condition and recover quickly, and is very suitable for day surgery. Since the establishment of the Glaucoma Treatment Center and day ward at Zhongshan Ophthalmic Center (ZOC), Sun Yat-sen University, peripheral iridectomy, trabeculectomy, glaucoma valve implantation, and other ambulatory operations have been developed. The current day surgery rate accounts for 60% of the total number of glaucoma surgeries [9].

Although the effects of the two hospitalization patterns on intraocular pressure (IOP) control have been compared [10, 11], the psychological status and mastery of knowledge among patients of the two patterns of hospitalization have never been investigated.

The purpose of this study was to compare anxiety and depression levels and glaucoma knowledge between glaucoma day-case patients and inpatients.

## 2. Methods

### 2.1. Study Population

Patients were enrolled from the Glaucoma Treatment Center of ZOC. All participants provided written informed consent in accordance with the Declaration of Helsinki. This protocol was approved by the ethics committee of ZOC (number: 2017KYPJ069) and is registered at clinicaltrials.gov (number: NCT03125850).

Inclusion criteria were as follows: (1) first admission to the Glaucoma Treatment Center; (2) diagnosis of glaucoma; (3) first time receiving glaucoma surgery; (4) voluntary participation in this study; (5) adequate language comprehension ability; (6) best-corrected visual acuity of 0.1 or more; and (7) informed consent signed by the patient or legal representative.

The exclusion criteria were (1) mental disorder; (2) visceral function failure or other serious diseases, including clinically related coronary artery disease, cardiovascular disease, or myocardial infarction experienced within the past six months; serious neurological or psychiatric illness; serious infections; abnormal coagulant function; general active infectious diseases; malignant tumour; or serious immune diseases; (3) monocular blindness; (4) other serious eye diseases; (5) participation in any clinical study within 3 months prior to enrollment; (6) perception by researchers that patient is not suitable for participation in this clinical trial; or (7) refusal to sign the informed consent form.

### 2.2. Research Instrument

#### 2.2.1. General Condition Questionnaire

The custom-made questionnaire included the participant's name, sex, marriage, profession, educational level, financial circumstances, systemic medical history, other ocular history, family history, and hospitalization times.

#### 2.2.2. Hospital Anxiety and Depression Scale (HADS)

The HADS [12] was developed by Zigmond and Snaith in 1983 as a self-report questionnaire designed to identify and quantify depression and anxiety among patients in nonpsychiatric hospital clinics. It consists of two subscales: HADS-anxiety (HADS-A) and HADS-depression (HADS-D). Each item is scored from 0 to 3, with higher scores indicating a higher level of depression and anxiety. For each of the seven-item subscales, the minimum sum score is 0 and the maximum is 21. In this study, we used the validated Chinese version of the HADS [13]. Patients with scores above 10 on the HADS-A or HADS-D were diagnosed with anxiety or depression, respectively.

#### 2.2.3. Gray Glaucoma Knowledge Questionnaire (GGKQ)

The GGKQ [14] was developed by Gray et al. in 2010 for patients with glaucoma. The questions cover the following topics: cause and effect of glaucoma, family history as a risk factor, testing, treatment, and follow-up. This questionnaire has a total maximum score of 17, with higher scores representing better knowledge.

### 2.3. Design

Sample size estimation: To calculate the sample size necessary for researching the differences in anxiety and depression between day-case patients and inpatients, with an estimated difference between the two population means of 0.5 times the standard deviation, a 1 : 1 sample size, a two-sided significance of 0.05, and a power of 0.8, it was necessary to enroll 86 patients. To determine the difference in glaucoma knowledge, assuming it has clinical significance when the difference between the two population means is 2 and with a sample standard deviation of 2.9 according to the previous study [15] and 1 : 1 sample size, 44 patients were required. To compensate for nonevaluable patients, we planned to enroll 120 patients per group.

The patients were randomized into 2 groups, the day-case group and the inpatient group, between August and October 2017. Three questionnaires including the General Condition Questionnaire, the HADS, and the GGKQ were distributed to the participants. All participants were asked to complete the three questionnaires at admission and the latter two at discharge.

All patients underwent comprehensive ophthalmologic examinations at admission including best-corrected visual acuity (BCVA), slit-lamp biomicroscopy, tonometry, indirect ophthalmoscopy, and visual field examination. BCVA was evaluated with Snellen equivalents, based on a standard refraction and testing protocol at a starting distance of 5 m. Intraocular pressure (IOP) was measured using a Goldmann applanation tonometer. Mean deviation (MD) was determined with automated perimetry using a 24-2 threshold program (HFA 24-2) with the SITA Standard strategy.

**Figure 1:**
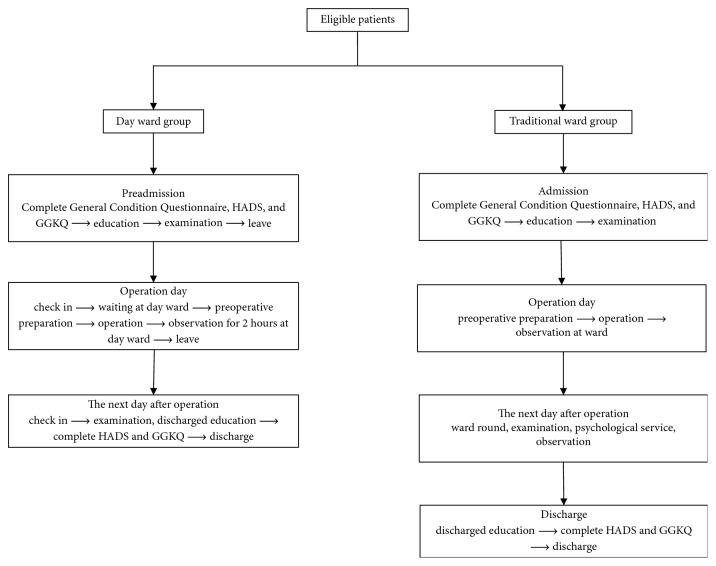
Flow diagram of procedures the patients went through. HADS, Hospital Anxiety and Depression Scale; GGKQ, Gray Glaucoma Knowledge Questionnaire.

The two groups underwent corresponding procedures for treatment, care, and education. A summary and flow diagram of the study design is provided in Figure 1.

### 2.4. Statistical Analysis

All statistical analyses were performed using IBM SPSS Statistics, version 20 (IBM Corp., Armonk, NY, USA). The research methods comprised descriptive statistical analysis, the two independent samples *t*-test, and the chi-square test.

## 3. Results

**Figure 2:**
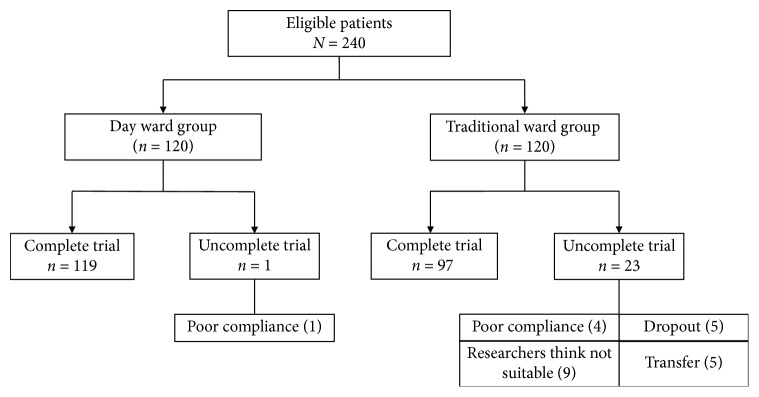
Patients followed throughout the study.

A total of 216 patients were ultimately enrolled in this study (Figure 2).

Table 1 shows the patients' major demographic and clinical characteristics. There were no significant differences between the two groups in terms of demographic and clinical characteristics, such as age, sex, and education (*P* > 0.05). However, the mean lengths of hospital stay for the day ward and inpatient groups were 1 and 3.07 ± 2.30 days, respectively.

**Figure 3:**
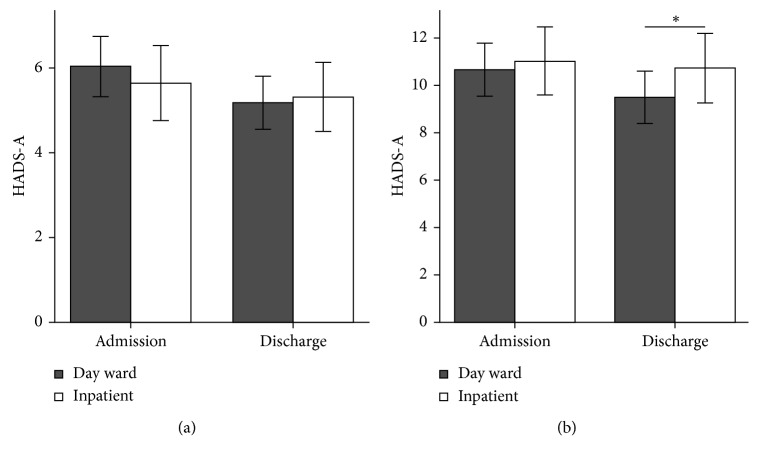
Comparison of anxiety and depression between the 2 groups using the HADS. HADS, Hospital Anxiety and Depression Scale; HADS-A, HADS-anxiety; HADS-D, HADS-depression. ^∗^*P* < 0.05. Error bars: 95% confidence intervals.

The mean HADS-A scores at baseline for the day-case patients and inpatients were 6.04 ± 3.92 and 5.65 ± 4.38, respectively; the mean scores before discharge were 5.18 ± 3.46 and 5.32 ± 4.04, respectively. The differences between the two groups were not significant (*P* > 0.05). The mean HADS-D scores for the day-case patients and inpatients at baseline were 4.60 ± 3.40 and 5.36 ± 3.59, respectively; no significant difference was observed (*P* > 0.05). However, the mean HADS-D score for the day-case group was 4.30 ± 3.33 before discharge, which was significantly lower than that of the inpatient group (5.39 ± 3.94, *P* < 0.001) (Figure 3).

**Figure 4:**
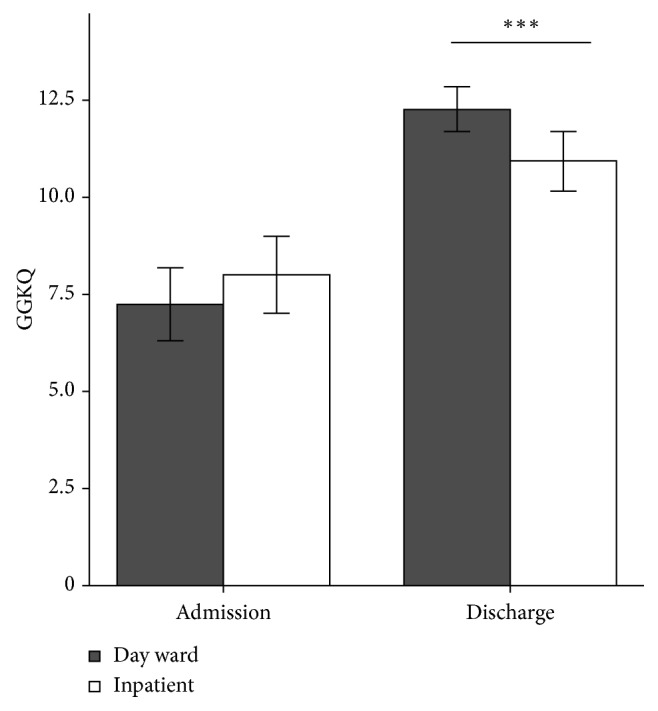
Comparison of glaucoma knowledge between the 2 groups. GGKQ, Gray Glaucoma Knowledge Questionnaire. ^∗∗∗^*P* < 0.001. Error bars: 95% confidence intervals.

For the GGKQ, the mean baseline scores were similar for the two groups: 7.24 ± 5.17 and 8.00 ± 3.29, respectively. However, the day-case patients' scores (12.27 ± 3.29) were significantly higher than the inpatients' scores (10.92 ± 3.79) before discharge (*P* < 0.001) (Figure 4).

## 4. Discussion

Glaucoma is a physical and mental disease that can potentially result in blindness and often causes psychological disorders. Previous studies have shown that the prevalence rates of anxiety and depression in patients with glaucoma are higher than the levels in the general Chinese population (anxiety: 2.4% and depression: 1.4%) [16]. Moreover, depression has been found to be associated with patients' perception of their vision [2]. The same study showed that depressive symptoms in patients are associated with faster visual field progression [17]. Day ward care as a patient-centred, quick, and convenient medical model that provides a level of care between outpatient and inpatient care has been gradually applied in the treatment of glaucoma patients in China. The safety and efficacy of day glaucoma surgery have been verified; a previous study found that there were no significant differences between inpatients and outpatients in terms of mean IOP or complications three months after surgery [10]. However, few studies have investigated differences in psychological factors and knowledge between day-case patients and inpatients; to the best of our knowledge, this is the first study to do so.

In our study, the overall prevalence rates of anxiety and depression in patients with glaucoma at baseline were 15.3% and 5.6%, respectively. These results are slightly different from those of previous studies. The occurrence of anxiety and depression differs greatly from region to region. In Japan, the respective prevalence rates were 13.0% and 10.9% in POAG patients [3]; in Turkey, they were 14.0% and 57.0%, and in America, 10.9% of glaucoma patients suffered from depression [18]. In Australia, the depression prevalence increased with glaucoma severity across all groups. The proportions of depressed individuals per group were 11.4% for mild glaucoma, 20.9% for moderate glaucoma, and 32.1% for severe glaucoma [19]. In China, Kong et al. reported anxiety and depression prevalence rates of 11.2% and 26%, respectively [20], and they were 22.92% and 16.40%, respectively, in the study conducted by Zhou [21]. The reason for these regional variances may be the different constituent ratios of glaucoma and the inclusion of patients of different races with different disease courses. At the same time, we found that although the baseline anxiety and depression levels were similar in both groups, the HADS-D scores of day-case patients were lower than those of inpatients (*P* < 0.001) before discharge. The results demonstrated that day-case patients were less depressed than inpatients before discharge. The reason may be that the patients were familiar with the home environment and felt relaxed at home. A hospital is not as familiar and comfortable as home, and this may increase the patient's negative emotions. It is a consistent wish of older people to remain at home because they feel attached to it [22]; this is particularly true for those with low vision. An enabling, safe, and comfortable environment may reduce particular stressors and encourage the individual to use available competencies [23].

Satisfactory treatment for glaucoma is dependent on the patient's self-care and compliance with the doctor's recommendations. Furthermore, patients' glaucoma knowledge is highly involved in both treatment and adherence. However, patients with glaucoma may have a lack of glaucoma knowledge and may experience psychological disturbance [3, 19]. In this study, the two groups were similar in baseline knowledge (*P* > 0.05), but the day-case patients' scores were higher than the inpatients' scores before discharge (*P* < 0.001). The mean number of hospital days of the day ward and inpatient groups was 1 and 3.07 ± 2.30, respectively. Although the hospitalization time for the day ward patients was 1 day, the interval between completing the questionnaires was 2 days, similar to that of the inpatients (*P*=0.785). Therefore, we can conclude that the day-case patients acquired more knowledge than the inpatients after education. The discrepancies may have occurred because the day-case patients were not as depressed as the inpatients. This finding agrees with a previous study that indicated that the level of glaucoma knowledge was negatively correlated with depression [20, 24]. Another reason may be that the day-case patients were more motivated because they knew that they had to take care of themselves without help from hospital workers after discharge [25].

The main limitations of our study included the relatively small sample size and short length of follow-up. Although there was no significant difference in sex, MD, or IOP, the male/female ratio was lower in the inpatient group, the MD of the worse eye was almost significantly lower, and IOP of the worse eye was slightly higher. These inconsistencies at baseline might also have contributed to the difference between the two groups. These results need to be confirmed in a larger sample size with a longer follow-up period.

In conclusion, our findings indicate that day-case patients have lower depression levels and higher glaucoma knowledge levels than inpatients. It is suggested that day ward care can be extended to suitable patients to reduce mental pressure and enhance glaucoma knowledge.

## Figures and Tables

**Table 1:** Demographic features and clinical characteristics of all participants.

	Day ward (*n* = 119)	Inpatient (*n* = 97)	*P*
Age (y)	52.26 ± 15.87	54.49 ± 15.35	0.297
Sex			
Male	50	47	0.344
Female	69	50	
Education			
Primary school	18	17	0.459
Junior high school	27	28	
High school	49	30	
University and above	25	22	
Marital status			
Single	13	7	0.421
Married	105	90	
Divorced/separated/widowed	1	0	
Hospitalized times	1.04 ± 0.20	1.06 ± 0.43	0.655
Hospital day	1	3.07 ± 2.30	<0.001
Duration of glaucoma (y)			
<1	71	57	0.774
1∼5	33	28	
5∼10	7	8	
>10	8	4	
Type of glaucoma			
PACG	63	61	0.520
POAG	26	22	
Mixed glaucoma	3	2	
Secondary glaucoma	20	9	
Congenital glaucoma	4	2	
Others	3	1	
Medical insurance			
Yes	66	55	0.964
No	53	42	
MD of HFA 24-2 (dB)			
Better eye	−7.21 ± 8.46	−10.0 ± 9.72	0.052
Worse eye	−17.45 ± 10.62	−19.8 ± 9.85	0.164
IOP at baseline (mmHg)			
Better eye	15.26 ± 6.40	16.32 ± 6.86	0.242
Worse eye	22.84 ± 11.81	25.87 ± 12.17	0.066
Visual acuity			
Better eye	0.65 ± 0.33	0.58 ± 0.40	0.260
Worse eye	0.35 ± 0.33	0.26 ± 0.26	0.053

PACG, primary angle-closure glaucoma; POAG, primary open-angle glaucoma; MD, mean deviation; IOP, intraocular pressure; dB, decibels.

## Data Availability

The Excel spreadsheet including the data is available from the corresponding author upon request.
